# Honey bee nutritranscriptomics reveals key insights towards precision nutrition

**DOI:** 10.1016/j.jare.2025.09.050

**Published:** 2025-09-30

**Authors:** Alexander McMenamin, Vincent Ricigliano

**Affiliations:** a USDA-ARS Honey Bee Breeding, Genetics, and Physiology Research Unit, Baton Rouge, LA 70820, United States; b USDA-ARS Pollinator Health Unit, Davis, CA 95616, United States

**Keywords:** Honey bee, Nutrition, Microalgae, Nutrigenetics, Nutritranscriptomics, Precision nutrition

## Abstract

**Introduction:**

Honey bees are essential managed pollinators faced by nutritional deficiencies which contributes to worldwide colony losses. The integration of nutrition science with genomics offers a powerful approach to optimize honey bee health by augmenting immune function and resilience to stressors through precision diet interventions.

**Objectives:**

To uncover the impact of honey bee genetics on core physiological responses to natural and artificial diets.

**Methods:**

Two honey bee genetic backgrounds (Russian and Pol-line) were fed diets consisting of sugar, pollen, and microalgae biomass from either *Chlorella vulgaris* or spirulina. Nutrigenetic variation was assessed by body weight measurements followed by transcriptome sequencing. Standard differential gene expression analysis and supervised machine learning algorithms were used to measure genotype-specific transcriptional responses to the diets.

**Results:**

Known (i.e., *vitellogenin* and *Jhe, hex70a*) and novel (i.e., *Glob1, slif* and *sad*) biomarkers of nutritional health exhibited a significant genetics-by-diet interaction. Furthermore, Russian bees featured a contracted transcriptional response, and a transcriptomic signature of delayed behavioral maturation as compared to Pol-line bees, suggesting alternative nutrient assimilation strategies between the two genetic backgrounds. Regardless of diet, Russian bees had significantly lower abundance of a major viral pathogen (DWV). Lastly, higher expression of humoral and cellular immune genes was detected in microalgae-fed bees, though spirulina was significantly more immunogenic than *Chlorella*.

**Conclusion:**

Genetic background plays a key role in shaping honey bee responses to nutrition. By identifying novel biomarkers of nutritional status, our results support the unique benefits of two microalgae species as functional feed additives. Furthermore, these results reveal the importance of genetics as a consideration when optimizing supplemental feed for an agricultural pollinator. These findings contribute towards the development of tailored nutrition strategies and artificial diets to improve honey bee health.

## Highlights

• Genetic background is associated with unique transcriptional and physiological responses to nutrition.
• Genetic stocks may utilize alternative social nutrient assimilation strategies.
• Novel molecular biomarkers were established to inform precise diet quality assessment.
• Russian bees had significantly lower abundance of DWV, a major pathogen threat to honey bee health.
• Two microalgae species have complementary benefits as functional feed additives.

## Graphical abstract

**Figure:**
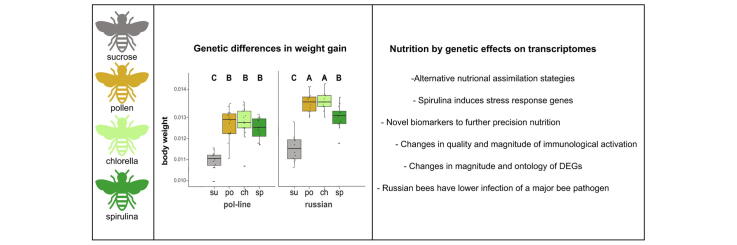

## Introduction

The interplay between the genome and nutrition is remarkably complex. Advances in next generation sequencing have greatly facilitated metabolic network reconstruction to assess how organisms acquire and process nutrients [1]. However, predicting the effects of genetic mutation on protein function, let alone metabolism, remains a major computational hurdle [2]. Using ‘Omics data in conjunction with dietary manipulations has proven to be a powerful approach to understanding phenotypic differences in nutritional integration [3]. Applying those insights to augment the prevention and management of disease is the essence of the field of nutrigenetics [4]. The application of nutrigenetics to identify the benefits of food components has grown particularly fast in recent years [5]. For example, phytonutrients have been shown to be a rich source of immunomodulatory compounds for humans and livestock [[6], [7], [8]].

The western honey bee (Apis mellifera) is the premier invertebrate livestock species and crop pollinator in the US and Europe. As such, ensuring honey bee colony health and productivity is paramount for global food security [9,10]. However, land-use change such as agricultural intensification has resulted in a depauperate floral landscape, thereby limiting the diversity and quality of pollen available to pollinators [[11], [12], [13]]. At the same time, pathogens pose a serious risk to honey bee colonies, which is exacerbated by poor nutrient availability [[14], [15], [16]]. Pollen-substitutes are artificial diets derived from alternative protein sources − such as soy lecithin − supplied by beekeepers to offset colony nutritional deficiencies, but these diets often fail to recapitulate the nutritional and immune benefits of pollen-based diets [16,17]. Therefore, there is an urgent need for sustainable feed additives that are at least as nutritious as pollen and that can provide functional benefits beyond their macro- and micronutrient content.

Microalgae have shown promise as functional feed additives in a variety of organisms [18], including honey bees [[19], [20], [21], [22]]. Microalgal biomass is a particularly attractive candidate for sustainable nutrient production since it requires less resources than terrestrial farming [23]. At least two species (*Chlorella vulgaris* and *Arthrospira platensis*) are comparable to pollen in terms of their nutrient content and bioavailability in honey bees [[20], [21], [22]]. Metabolomic analyses previously revealed that the lipid-rich biomass of *Chorella* may make it nutritiously comparable to pollen [20] and proteomic analyses indicated that spirulina upregulates expression of select nutritional and immune-related transcripts [21]. Furthermore, microalgae are amenable to genetic manipulation and can serve as production and delivery platforms for honey bee antiviral therapeutics [24]. Feasibly, this could be stacked with essential pollen sterols like those recently produced in genetically engineered, nutritionally enhanced yeast [25]. Collectively, we are on the precipice of revolutionizing sustainable colony management through modern biotechnological practices (reviewed in [26]). Thus, an increased understanding of the effects and benefits of microalgae-containing feed across genetic backgrounds will aid in functional feed development for managed bees.

Here, we aimed to better understand the physiological and transcriptomic impacts of natural and artificial diets in two genetic lines of Varroa mite resistant honey bees, each maintained as closed breeding populations – the Pol-line [27] and Russian bees [28,29]. These lines are genetically distinct with differing responses to nutrition. For example, a study on the effect of diet on *vitellogenin* expression reported that Russian bees were significantly more sensitive to protein starvation [30]. Additionally, a previous nutrigenetic comparison of Pol-line and Russian bees found that while, overall, Spirulina supplementation led to heavier bees, Pol-line bees accumulated more abdominal lipids and heavier thoraces [22]. In fact, these individual-level physiological differences are also associated with gross colony behavior differences, as Pol-line colonies maintain a higher cluster temperature than Russian bees, although Russian colonies consumed less food [31]. Therefore, these two stocks have distinct physiologies that make them good comparison points for nutrition studies.

Therefore, we hypothesized that bees from these two distinct genetic lines (henceforth referred to as “genetic backgrounds”) would have different physiologic and transcriptional responses to diets composed of pollen or microalgae. To test this, we assessed body weight and transcriptome-wide changes in two honey bee genetic stocks (Russian and Pol-line) fed four different diets: 1) carbohydrate only (i.e., sucrose), 2) pollen 3) *Chlorella* or 4) spirulina biomass. We leverage this nutritranscriptomic experimental design to identify novel molecular biomarkers and describe a transcriptional signal of alternative social nutrient assimilation strategies between these two commercial stocks. Further, our analyses demonstrate that spirulina effectively induces the expression of stress- and immune-response genes, and therefore may improve pathogen resilience [32]. On the other hand, *Chlorella* is less immunogenic but may be exceptionally nutritious – rivalling even pollen. Altogether, this study reveals key insights toward precision nutrition for honey bees, the premier agricultural pollinator for the US and Europe.

## Materials and methods

### Honey bee cage assays and physiological measurement

Hives used for sourcing study organisms were maintained using standard apicultural practices in Baton Rouge, Louisiana with routine Apivar treatment to control Varroa mite levels. One day before experimental set-up, capped brood frames were transferred from 3 Russian and 4 Pol-line parent colonies to a humidified incubator maintained at 35 °C and 50 % relative humidity. Parent colony was recorded and used as a blocking variable during the experiment described below. The following morning, groups of 50 age-matched newly emerged worker bees (*Apis mellifera*) were collected into hoarding cages which were then randomly assigned a treatment group. Each treatment group (sugar, pollen, *Chlorella* or spirulina) was replicated across 4 cages for a total of 48 Russian replicates and 64 Pol-line replicates. Bees were fed sucrose syrup (50 % w:v) and their assigned diet *ad libitum* for 8 days. At the end of the study, bees were collected onto dry ice and dissected into head, thorax (excluding wings and legs), and abdomen and collected into pools of 8 body segments per cage. Abdomens were collected into 2 mL homogenization tubes charged with 1.4 mm ceramic beads and stored at −80 °C until nucleic acid extraction for further analysis. In separate Eppendorf tubes, heads and thoraces were dried at 60 °C for 48 hrs and weights were measured to the nearest 0.1 mg as a proxy for nutrient integration. For analysis, the total weight of each pool of body segments was divided by 8 to get the average body segment weight per bee. Sample metadata and raw body weight data can be found on Table S1.

### Diet preparation and consumption measurement

Diets were prepared as previously described [20]. Briefly, powdered diet components (sugar, corbicular pollen, *Chlorella*, or spirulina) were mixed 1:1 (v:v) with sucrose syrup to a paste consistency and delivered in modified microcentrifuge tubes. Evaporation-controlled diet consumption was recorded on days 4 and 8, as described previously [20]. Cumulative weight of consumed diet was used for analysis in this study (Table S1). *Chlorella* and spirulina powders used for diet formulation were nutritionally comparable to the bee-collected pollen used as evidenced by amino acid content (Table S2).

### Transcriptomics

Pooled abdomens were homogenized using a Bead Rupture Elite bead mill (OMNI International, Kennesaw, GA, USA) on speed 4 for 30 s and total RNA was isolated with a Monarch total RNA miniprep kit (New England BioLabs) according to the manufacturer’s instructions and subjected to DNase treatment. 1 μg of total RNA was sent to the Roy J. Carver Biotechnology center for library preparation and transcriptome sequencing. The RNAseq libraries were prepared with Kapa Hyper Stranded mRNA library kit (Roche), which used poly-A selection for mRNA enrichment of libraries. The libraries were pooled, quantitated by qPCR and 2 x 150 nt reads were sequenced on two S4 lanes for 151 cycles from both ends of the fragments on a NovaSeq 6000. Fastq files were generated and demultiplexed with bcl2fastq v2.20 Conversion Software (Illumina). FastQC was used to remove low quality reads (<Q30) and 3′ end adaptors were removed by trimming the first 33 nt.

Reads were aligned to a reference consisting of the *Apis mellifera* HAv3.1 genome assembly and 5 viral genomes (DWV A, DWV B, ABPV, IAPV and sacbrood) using STAR (v2.7.9a) and uniquely mapped reads were counted with featureCounts (Subread v2.0.2) using default parameters. On average, 88.95 % of the ∼ 12.3 billion reads across 112 libraries were uniquely mapped. See Table S3 for library mapping information (e.g., total reads and mapping rates) and Table S4 for raw transcript read counts. Trimmed sequencing data was deposited into the NCBI Sequence Read Archive under BioProject number PRJNA1248567.

### Statistical analysis

**Fig. 1:**
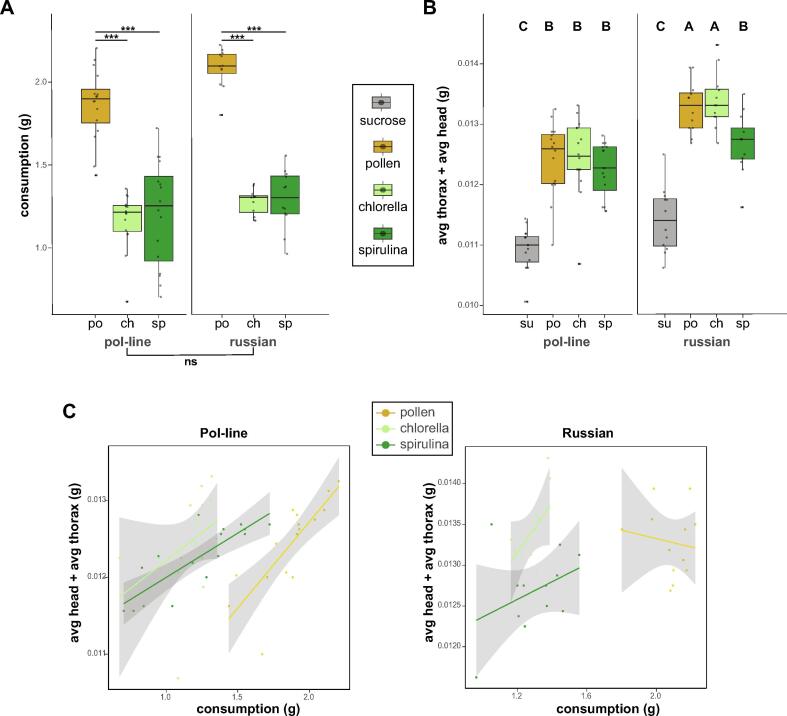
**Genetic variation in physiologic response to diet.** (A) Consumption weights are presented as the sum total of food consumed at days 4 and 8 of the experiment. Both algae-substituted diets were consumed equally to one another and significantly less than pollen. There was no difference in consumption between stocks (Linear mixed model, *p < 0.05, **p < 0.01, ***p < 0.001). (B) Bees fed protein/lipid-based diets are heavier than the sucrose-fed controls. Spirulina-fed Russian bees, but not Pol-line, have lower head and thorax weights than *Chlorella* and pollen fed-bees. Lastly, on average, Russian bees are heavier than Pol-line bees (Linear mixed model; The connecting letters report along the top of each boxplot indicates statistical differences.). (C) Regardless of diet, greater food consumption was associated with higher dry body mass. See Table S2 for full statistical model outputs.

Analyses were conducted and figures were generated in R version 4.4.0 “Puppy Cup” [33]. Linear model fixed and random effect structures were determined *a priori* with no subsequent model selection procedures to test specific hypotheses. Simple linear regression models were constructed using the base R *stats* package, and mixed effects models were fit using the lme4 package [34]. *Post hoc* analyses of linear models were performed by calculating estimated marginal means of group differences using the *emmeans* package (v 1.10.5) [35] Transcriptome analysis was performed with the DESeq 2 (v 1.44.0) and *limma* packages. The MDS plot in Fig. 3 was generated using the *plotMDS* function native to *limma*, in pairwise mode which identifies the top 300 transcripts with the largest expression difference between any two given samples to maximize the Euclidean distance between points. For details on statistical analysis of sequencing data see below sections. Raw output from statistical analysis of diet consumption and body weight presented in Fig. 1 can be found on Table S5.

### Differential gene expression and gene ontology analysis

Prior to statistical analysis transcripts with fewer than 50 total reads across all libraries were removed and transformed using the varianceStabilizingTransformation function in the DESseq2 package (v 1.44.0) [36]. Normalized transcript counts showed uniformity of distribution across all 112 libraries (Fig. S1). *Limma-voom* [37] and empirical Bayes error moderation were used to calculate differential expression of transcripts in pollen, *Chlorella*, and spirulina fed groups with sugar-fed bees as the reference level and colony source as a blocking variable (Table S6 and S7). Contrasts of interest (e.g., spirulina-fed versus pollen fed pol-line bees) were specified manually using the *makeContrasts* function in *limma* and, again, empirical Bayes moderation of test statistics was used to assess differential transcript expression (Table S6 to S8). Gene ontology analysis was carried out using ShinyGO 0.8 online tool [38] using the annotated *A. mellifera* DH4 Amel_HAv3.1 chromosome level assembly and with list of genes remaining after filtering lout low-read count transcripts as the background, unless otherwise specified.

### Random Forest regression

**Fig. 2:**
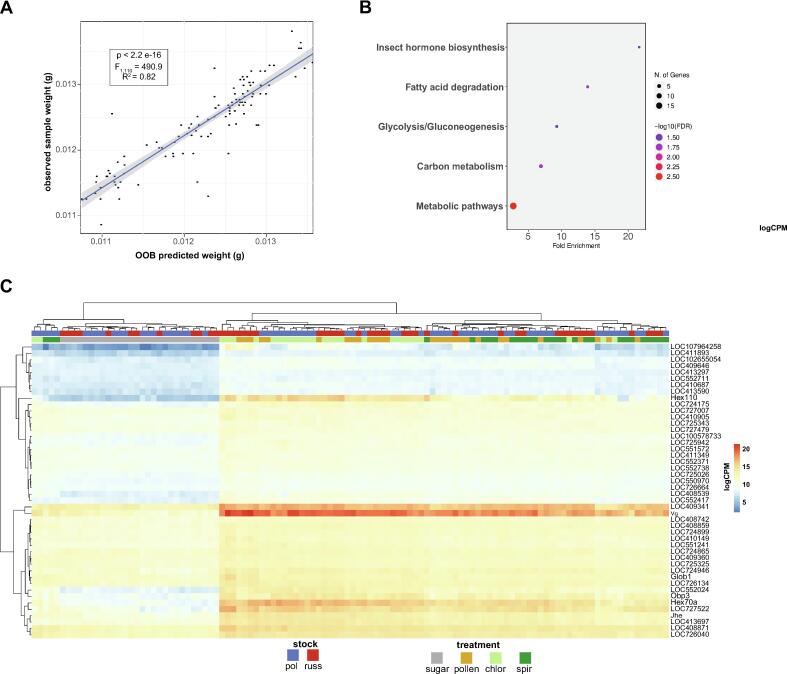
**Random Forest Regression identified known and novel biomarkers that predict body mass.** (A) The full dataset −including all diets and both stocks- was utilized to iteratively train RF models to recursively select for themost informative features (Table S9, S10). The optimal 308 gene regression model accurately predicts bee body mass based on normalized transcriptomes (predicted weight) as compared to observed body mass. (B) Of those 308 features, 172 of them are positively correlated with body weight. These are enriched for genes associated with hormone synthesis, and metabolic pathways (Table S11)(C) Normalized transcript counts of genes positively correlated with body weight were plotted as a heatmap which shows clear elevated expression in PL-fed bees relative to sugar-fed bees. Overall, these biomarkers indicate that Chlorella may be a particularly good source of protein and lipids for bees. Additionally, *Jhe, drop dead* and *Glob1* are outlined in red as they behaved most similar to expectations, as spirulina-fed Russians had notably lower expression.

Random Forest regression is an ensemble learning method that uses multiple decision trees to make a more stable prediction of your response variable given a list of features. Due to the ensemble nature of Random Forest models, they tend to perform well on datasets with more features than samples, especially when feature selection is applied [39]. In this case, a Random Forest regression model was implemented using the *ranger* package in R [40] to predict the sum of the average head and thorax weight measured per cage as a function of normalized transcript abundance. After filtering for low abundance transcripts and applying a variance stabilizing transformation, Recursive Feature Elimination was used to select transcripts with the highest relevance to body weight. To do this, an initial Random Forest Regression model was fit to the full dataset with 11,310 transcripts included as predictors of body weight. Then, transcripts which negatively impacted model performance as measured by corrected variable importance score were removed. The resultant reduced set of transcripts was then used to tune a subsequent RF regression model. This procedure was repeated iteratively until the optimal model structure was found by Out-of-bag (OOB) RMSE minimization (Fig. S2). The optimal model included 308 features. The dry weights of OOB samples were predicted using the optimal random forest regression model to assess model performance (Fig. 2A). Then, the Pearson correlation coefficients between normalized read count and sample weight were calculated for the top 308 features (Table S10). Features with significant, positive correlations with body weight were subjected to Gene Ontology analysis. Lastly, an arbitrary cutoff of *r =* 0.75 was used to select a final set of 46 biomarkers for body weight.

### Pol-line versus Russian-defining feature selection using Support Vector Machine learning

Support Vector Machines are a supervised classification algorithm which we used to classify bees as being Pol-line or Russian based on the transcript count matrix following variance stabilizing transformation. To initially reduce the number of possible features, we selected the 2,914 differentially expressed genes shared by both stocks regardless of diet. Then, we used the sigFeature recursive feature elimination algorithm with a linear kernel [41] to select features with maximum discriminatory power between classes followed by ten-fold external cross-validation to assess SVM model performance, which showed significant reduction of external cross-validation error and model standard deviation (and therefore high model performance) with as few as the top 20 genes (Fig. S3, Table S11). Unsupervised hierarchical clustering of samples by Pearson correlation of normalized gene expression was used as a naïve *post hoc* assessment of the features selected by the SVM model (Fig. S4).

## Results

### Honey bee stocks vary in weight gain under different dietary conditions

Newly emerged, age-matched Pol-line and Russian workers were subjected to one of four diet regimes: (1) no protein diet (sugar only), (2) pollen, (3) *Chlorella* or (4) spirulina. Throughout the manuscript, diets 2, 3 and 4 will collectively be referred to as a protein/lipid containing diets (PL diets). Diet consumption and weight data were measured at the end of the study as whole-cage physiology metrics.

Bee stock had no effect on diet consumption, however both stocks consumed less of the microalgae diets relative to pollen (linear mixed model; Fig. 1A, Table S5). Algae diets resulted in weight gains comparable to pollen diets despite lower consumption (linear mixed model; Fig. 1B, Table S5). While Pol-line and Russian bees fed only sugar weighed the same on average, pollen- and *Chlorella*-fed Russian bees were heavier than their pol-line counterparts (linear mixed model with post hoc pairwise comparisons, pollen: t = 2.7, q value = 0.046; *Chlorella*: t = 2.9, q value = 0.036). Interestingly, when fed spirulina, there was no difference in the average weight of pol-line and Russian bees. In fact, spirulina-fed Russian bees are lighter on average than those fed pollen (t = 3.82, q value = 0.0006) or *Chlorella* (t = 4.52, q value = 0.001). Regardless of diet, increased food consumption was correlated with higher head and thorax weight (linear mixed model, R^2^_m_ = 0.46, R^2^_c_ = 0.69; Fig. 1C, Table S5).

### Identification of novel biomarkers by Random Forest machine learning

Next, we sought to infer nutrition-related biomarkers from our dataset. Recursive Feature Elimination was applied via iterative Random Forest regression models to identify the optimal number of features to predict bee body weight [39]. Ultimately, the model with the lowest OOB error included just 308 transcripts (R^2^ = 0.809, OOB RMSE = 0.000400885, Table S9, Fig. S2). Together these features predicted sample weight for out-of-bag samples (Fig. 2A; R^2^ = 0.82, F_1,110_ = 490.9, p < 2.2x10^-16^) with high accuracy. Since the goal was an expanded, but still pragmatic set of biomarkers, we calculated the correlation between transcript abundance and bee weight as an additional feature selection step. Of these 308 transcripts, 172 had a significant positive correlation with bee weight (see Table S10 for the full list of features and their correlation with body weight), which were enriched for genes involved in hormone synthesis, fatty acid degradation, glycolysis/gluconeogenesis, carbon metabolism and metabolic pathways (Fig. 2B, Table S11). Next, the set of putative biomarkers was further reduced to just 46 transcripts by applying an arbitrary cutoff of *r* = 0.75. It is clear from a heatmap of transcript abundance that sugar-fed bees have lower expression of weight-associated transcripts, on average. Furthermore, this approach identified *Vg*, a well-known biomarker for honey bee health [42], supporting the validity of this approach to identify bona fide biomarkers (Fig. 2C). For the purposes of this study, we chose six biomarkers with clear biological functions associated with metabolism and/or growth. Just six biomarkers were chosen for further interrogation in the context of the present study: 2 nutrient storage proteins (*Vg*, *Hex70a*), 2 insect hormone synthesis-related genes (*Jhe*, and LOC411893), 1 respiration-related genes (*Glob1*) and one fat body TOR signaling gene (LOC409646). We hypothesized that these biomarkers would have lower expression in spirulina-fed Russian bees, commensurate with their reduced weight gain (Fig. 1C),

Overall, sugar-fed bees had lower expression of all 6 biomarkers, an indication of their starvation state. As predicted, both nutrient storage proteins (*Vg* and *Hex70a*) and hormone biosynthesis genes (*Jhe,* and LOC411893, a.k.a. *sad*) had significantly lower expression in Russian bees fed Spirulina, as compared to their pollen and *Chlorella-fed counterparts* (Fig. S4). A bit more surprisingly, these 4 biomarkers also had significantly lower in spirulina-fed Pol-line bees compared to pollen and *Chlorella*-fed, as well (Fig. S3; see Table S12 for full statistical outputs). This may suggest that these biomarkers capture nutritional stress in the absence of an effect on gross metrics, like body weight. Interestingly, *Glob1*, a gene involved in insect respiration [43] is highest in *Chlorella*-fed bees, and especially Russian bees, but had similar expression in Pol-line bees fed pollen or spirulina (Fig. S4; Table S12). Lastly, higher expression of LOC409646 (akak, *slimfast*) in all PL diet fed bees with no difference between PL diets, suggests *slimfast* may more broadly indicate generally good nutritional status. Taken altogether, these results highlight the utility of the identified biomarkers to assess the quality and impact of nutritional supplementation across genetic backgrounds.

### Transcriptome similarity is primarily driven by starvation status and secondarily by genetic background

**Fig. 3:**
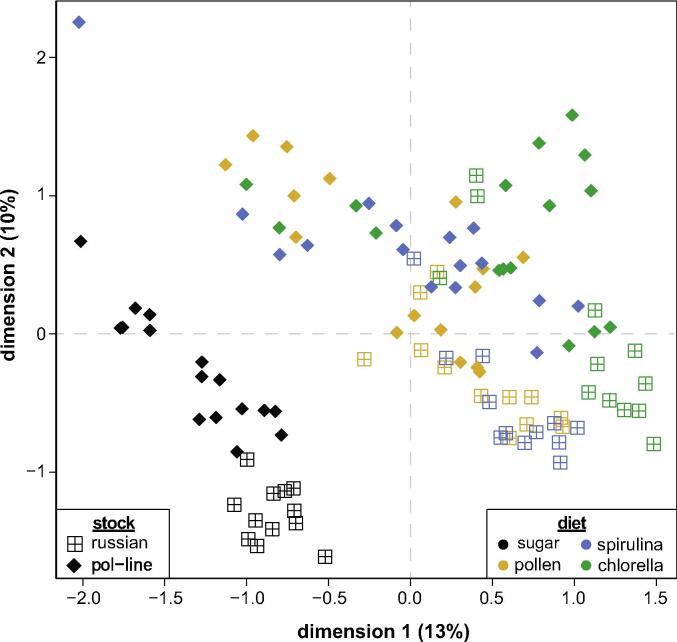
**Transcriptomic differences are driven primarily by diet and secondarily by genetics**. Dissimilarity between any two samples was calculated as the typical log_2_-fold change of the top 300 pairwise differentially expressed genes (DEGs), therefore the further two samples are from each other the greater the difference in transcriptome similarity. The majority of the variation in transcriptomes is apparent along the horizontal axis, which is driven by diet identified by color. Clear sample differentiation is also occurring on a diagonal from the top left to the bottom right, which is driven by genetic stock.

Multidimensional scaling of sample distances revealed that most variation in global gene expression was driven by whether bees received a PL diet (dimension 1 = 13 % of variation explained, Fig. 3). As the transcriptomes of pollen- and algae-fed bees cluster closer together when projected onto 2-dimensional space due to smaller sample-distances between points, they are more similar to each other than they are to bees fed only sugar (PL starved). The effect of stock on global gene expression is more apparent along the vertical axis, but explained a smaller amount of variation (dimension 2 = 10 % of variation explained, Fig. 3). Therefore, sugar-fed bees were used as the control group for calculating differential gene expression as they have the most distinct transcriptomes (Table 1).

**Table 1:** Number of genes differentially expressed between groups.

	Pol-line pollen	Pol-line *Chlorella*	Pol-line spirulina	Russian pollen	Russian *Chlorella*	Russian spirulina
Pol-line pollen	4190	5373	3385	126	na	na
Pol-line *Chlorella*		6814	730	na	451	na
Pol-line spirulina			5470	na	na	2709
Russian pollen				2468	3683	5765
Russian *Chlorella*					5208	3119
Russian spirulina						4672

Table 1**Pairwise DEG counts.** Cells at the intersection of a row and column of the same name represent the number of genes differentially expressed in that group relative to bees fed only sugar. Cells at the intersection between a row and column with different names represent the number of differentially expressed genes after calculating contrasts between those groups after applying limma-voom to calculate expression relative to bees which received only sugar.

### Bees fed a PL-diet have a core set of DEGs involved in metabolic and nutrient utilization as well as innate immunity

**Fig. 4:**
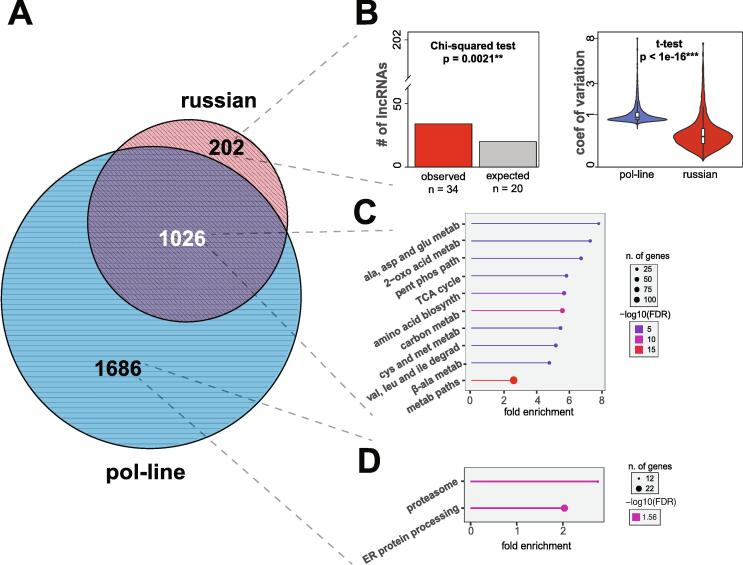
**Transcriptome similarities and differences between genetic stocks when fed any protein/lipid diet.** The Russian (1228 DEGs) and Pol-line (2712 DEGs) circles are the genes always differentially expressed in each respective stock when fed pollen, *Chlorella* or spirulina relative to sugar-fed bees. Then, those two DEG lists were overlapped to obtain this resultant Venn diagram. Unique Russian DEGs did not have significant ontological enrichment, but had significantly higher lncRNAs than expected. On the other hand, Pol-line DEGs were significantly enriched for genes involved in proteosome biology and ER processing.

Next, we assessed the transcriptional response to PL diets across Pol-line and Russian bees. To do this, the *limma* package was used to identify differentially expressed genes (DEGs) in pollen-, spirulina- and *Chlorella*-fed bees relative to sugar-fed bees using source colony as a blocking variable. Then, we took the intersection of the DEGs in bees fed the three diets, which identified 2,712 DEGs in Pol-line and 1,228 genes in Russians that were differentially expressed in all groups that received a PL diet. The two stocks shared 1,026 DEGs. These DEGs were involved primarily in small molecule metabolism, such as amino acids and glucose (Fig. 4A and B). There were also shared DEGs involved in nutrient storage (e.g., *vitellogenin*, *Hex70a, Hex110*), energy production (e.g., ATP5G2, *ArgK*), innate immunity (e.g., *B-gluc1, PGRP-S2* and *S3, apidaecin 1*). See Table S13 for full lists of shared and unique DEGs in Pol-line and Russian bees.

### Russian and Pol-line bees have altered transcriptional programs in response to nutritional perturbation

**Fig. 5:**
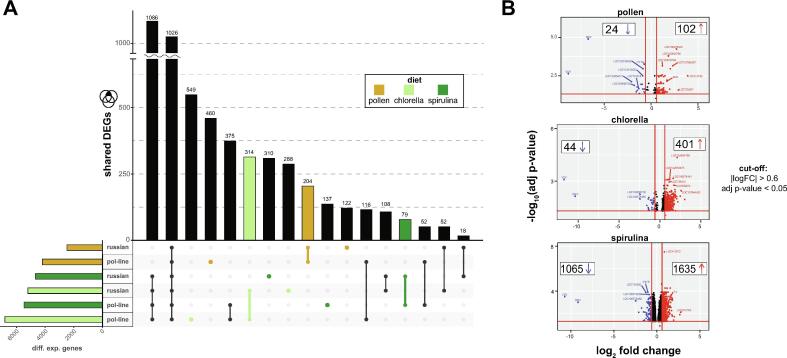
**Russian and Pol-line transcriptomes are most different when they are fed spirulina.** (A) The total number of DEGs calculated within diets by stock are indicated in the bar chart on the bottom left. Select intersections are indicated on the intersection matrix below the bar chart displaying the size of said intersection. (B) Contrasts were calculated between stocks within diets and visualized as volcano plots. Significantly differentially expressed genes (FDR < 0.05, log_2_ FC > 0.6) are indicated in red if they are higher in Russians and in blue if they are lower in Russian bees. Notably, DWV-A and DWV-B are consistently lower in Russian bees as compared to Pol-line bees.

Both Pol-line and Russian bees exhibited marked global transcriptional responses to PL diets with more than 2000 DEGs compared to sucrose-fed bees (Fig. 4A, Table 2). This is expected since sucrose-fed bees were experiencing a starvation response due to the absence of essential macronutrients. When contrasts are calculated between stocks, within diets, the most highly differentially expressed gene in Russians is consistently a very small protein of unknown function (LOC113218735), thought the majority of the top genes are either of unknown function or non-coding. Notably, Russian bees also had significantly lower DWV than Pol-line regardless of diet (Fig. 4B, Table S8). However, the most striking result was that Russian bees consistently had fewer DEGs than Pol-line bees and that both stocks had more DEGs when fed microalgae as compared to pollen (Fig. 5A, S7 and S8).

**Table 2:** Humoral and cellular immune genes chosen for investigation.

**Gene name**	**Accession**	**Function/pathway**	**Reference**
*defensin 1*	NM_001011616.2	Antimicrobial peptide	[117]
*apidaecin 1*	NM_001011613.1	Antimicrobial peptide	
*hymenoptaecin*	NM_001011615.1	Antimicrobial peptide	
*abaecin*	NM_001011617.1	Antimicrobial peptide	
*PGRP-S1*	XM_001121036.5	Pathogen recognition receptor	
*PGRP-S2*	NM_001163716.1	Pathogen recognition receptor	
*PGRP-S3; PGRP-SA*	NM_001163715.1	Pathogen recognition receptor	
*cactus 1*	NM_001163712.1	Toll pathway; NF-κB inhibitor	
*PPOact*	XM_001121888.5	Melanization	
*PPO*	NM_001011627.1	Melanization	
*Amel\102*	NM_001327953.1	Toll pathway; Melanization/encapsulation	[118]
*TEP7*	XM_006565440.3	Opsonization; cellular immunity	[119]
*TEPB; CD109 antigen-like*	XM_026441482.1	Opsonization; cellular immunity	[120]

Overall, transcriptional responses to diet were similar between stocks, though the relative overlap in DEGs is highest in *Chlorella*-fed bees (Fig. 5A, Fig. S8). Since Russian bees had fewer DEGs overall we hypothesized that they would also have differential expression of genes involved in transcriptional regulation and chromatin remodeling. To test this, we extracted DEGs shared among all diet groups within stocks (Fig. S7) and then assessed the overlap of those two lists (Fig. 4A).

Pol-line bees featured 1686 unique DEGs compared to Russians which were enriched in genes involved in proteasome activity, protein processing in the endoplasmic reticulum and macromolecule localization in the cell (Fig. 5A and B, and Table S14). Upregulated Pol-line DEGs were also enriched in transcriptional activators, DNA binding, RNA processing, RNA polymerase II transcription, and ncRNA processing (e.g., tRNA splicing machinery) (Table S14). In contrast, Russian bees had far fewer unique DEGs when fed any PL diet as compared to Pol-line bees – just 202. There were no significantly enriched GO terms, but categorization of transcripts for coding potential revealed that Russian bees had more differentially expressed long noncoding RNAs (lncRNAs) than expected (χ^2^ = 9.47, p = 0.002, Fig. 4B). Whereas categorization of Pol-line-specific DEGs demonstrated they had fewer differentially expressed lncRNAs than expected (χ^2^ = 5.5, p = 0.019, Fig. S6). Non-coding RNAs serve diverse functions, though lncRNAs are broadly implicated in transcriptional regulation and suppression. The smaller number of DEGs in PL-fed Russian bees is intriguingly associated with a lower coefficient of variation of transcript abundance (Fig. 4B, right panel; t = 46.448, df = 22127, p < 2.2x10^-16^). It is tempting to speculate, then, that higher differential expression of lncRNAs in Russian bees is associated with their smaller and less variable transcriptional responses. This result is underscored when we look specifically at pollen-fed bees.

Pollen-fed pol-line featured 2,178 unique DEGs, including those associated with nucleic acid binding and processing, ribosome biogenesis and maintenance of protein folding (Table S16), reflective of the marked transcriptional response to pollen. Comparatively, Russians have fewer than a quarter (456) the number of unique DEGs as Pol-line and the only enriched GO term was phosphofructokinase activity, a glycolytic enzyme (Table S16) which may be associated with higher weight gain in Russians. This is corroborated by decreased expression of starvation response genes (e.g., *akhr, dopR2*). Additionally, their contracted transcriptional response is marked by differential expression of translational control genes (e.g., *eif4g, LOC552206,LOC411907, LOC551133, LOC724373)* of two histones (e.g., H2A, H2B) and a CpG island-binding protein (*LOC408891*) as well as decreased expression of a polycomb-like gene (*pcl*), suggesting significant chromatin remodeling. To aid in identifying set of genes potentially driving these observed differences, we next employed a machine learning classifier to select features defining Pol-line and Russian bees.

### Transcripts which maximally differentiate genetics stocks suggest a signature of delayed maturation in Russian bees

A Support Vector Machine (SVM) model with 10-fold external cross validation was used to select a limited number of features from the union of Russian and pol-line DEG lists – 2,914 genes total. This supervised clustering technique aimed to identify a reduced set of genes that have maximum discriminatory power between classes (Russians and Pol-line) by removing redundant features. This solves the inherently sparse nature of high-dimensional data by reducing the feature set to meaningful predictors. The SVM classifier indicated that error reached its minimum at 400 features, which lacked significant ontological enrichment. Furthermore, the cross validation error of the classifier was significantly lowered at just 20 features (Fig. S4). Therefore, we chose the top 20 transcripts with maximum utility in differentiating the two stocks. Seven of the top 20 features are predicted to be lncRNAs, all of which had higher expression in Russian bees relative to Pol-line bees. Another 5 features are uncharacterized or of unknown function. We were unable to identify homologues with known functions by PSI-BLAST or HMMER searches. The remaining 8 features had a variety of predicted functions based on homology searches (Fig. S4, Table S11).

Two of the genes are particularly interesting due to their predicted functions in neurophysiology and synaptic activity (i.e., *RYamide receptor/LKR and PPK16*). *RYamide/leucokinin receptors* (*LKR*) have diverse roles in *Drosophila* neurophysiology, including gustation, nociception, and suppression of feeding behavior [44]. Both transcripts had higher expression in bees fed a PL diet, and over-all higher expression in Russian bees. Consistent with the prior observation that spirulina may be a sub-optimal diet for Russian bees, spirulina-fed Russians had lower expression of *RYamide receptor*, which may indicate they were not satiated (Fig. S6, Table S11 and S15). Similarly, pickpocket proteins (PPK) are amiloride-sensitive degenerin/epithelial Na^+^ channels with a wide variety of neural functions including proprioception, and water detection and reception, potentially through regulation of synaptic homeostasis [[45], [46], [47]]. In honey bees, the functions of *PPK* homologues remain elusive [48], and attempts to detect leucokinin peptides have so-far failed [49,50]. Therefore, the implications for stock differences in the expression of these genes is unclear. However, further investigation is necessary as they may be useful in future honey bee dietary studies.

Lastly, *juvenile hormone acid methyltransferase* (*JAHMT*) was significantly lower in Russian bees relative to Pol-line. However, feeding on PL diets consistently increased transcript expression in both stocks (Fig. S6, Table S11 and S15). JAHMT catalyzes the conversion of JH precursors into bioactive JH [51]. This mechanism is associated with behavioral maturation in honey bees, as increasing JH titers coincides with task switching from nurse behavior to forager behavior [52]. Therefore, a reduced expression of a critical JH biosynthesis gene like JAHMT may point to a delay in behavioral maturation in Russian bees.

### The transcriptomes of pollen-fed Russian bees further implies a signature of delayed maturation

Russian bees had 102 genes up-regulated and just 24 genes down-regulated relative to pol-line bees, indicating similar responses to a pollen-based diet (Table S8, Fig. 5B, Fig. S9, Table S8). Consistent with our previous observation, Russian bees had an enrichment in lncRNAs, both up (e.g., *LOC102654788, LOC100578799, LOC107965387*) and down (*LOC102656752*, *LOC107965714*) regulated. Many of the remaining genes encoded predicted proteins of unknown function (Table S8). As dynein motor proteins and endosome recycling are important for proper *PPK* ion channel distribution during dendritic growth in *Drosophila* [53], we hypothesized that transcripts serving this function would be differentially expressed in Russian bees. Russian bees had several upregulated genes that may implicated in endosome recycling, and/or lysosome function multivesicular body transport (e.g., *LOC100576540, LOC411506*, *LOC411602, LOC413489, LOC552447*). Since we also observed higher *PPK16* expression relative to pollen-fed Pol-line, this may suggest that our experiment captured a period of relatively intense mechanosensory neuron dendrite growth in Russian bees. Lastly, JHAMT (a positive regulator of JH titers) and *AKHR* expression were lower in Russians, suggesting a delay in energy mobilization and concurrent delay in the switch to a “forager” phenotype.

### Lighter body weight in spirulina-fed Russians is associated with a marked stress response

Spirulina-fed Russians had 5,765 DEGs relative to pollen, but only 4,672 relative to sugar-fed bees, while spirulina-fed Pol-line had 3,385 and 5,470 DEGs relative to pollen and sugar, respectively. Spirulina-fed pol-line and Russian bees had 3,584 shared DEGs in total (Fig. S9C). These were significantly enriched for genes involved in small molecule metabolism (e.g., TCA cycles, nucleotide and amino acid biosynthesis), but they are also enriched for antibacterial defense response genes (e.g., *hymenoptaecin*, abaecin), lysosome, phagosome, and peroxisome genes (Table S17). Altogether, this indicates an increase in stress response gene activity.

Gene-wise contrasts were calculated between Russian and Pol-line bees. This identified 2,709 DEGs (Fig. 5B, Table 1), which were primarily enriched in mitochondrial and ribosome homeostasis (Table S19). The set of genes involved in mitochondrial respiratory chain complex assembly (GO:0033108: 3.77 fold enrichment, FDR = 0.012) were all up-regulated in Russian bees, suggesting a higher metabolic rate. Russian bees also had significant enrichment in genes associated with cellular component disassembly such as autophagy-associated genes like *Atg17* (Table S18)*.* Additionally, gene-wise contrasts between spirulina- and *Chlorella*-fed Russians reveals an enrichment in genes involved in DNA damage and repair (GO:0006974: 1.85 fold, FDR = 1.71x10^-5^; GO:0006281: 1.9 fold, FDR = 1.9x10^-5^) and general cellular response to stress (GO:0033554: 1.7 fold, FDR = 0.0005; GO:0006950: 1.55 fold, FDR = 0.003; Table S18).

### Microalgae diets elicit genotype-specific cellular and humoral immune responses

**Fig. 6:**
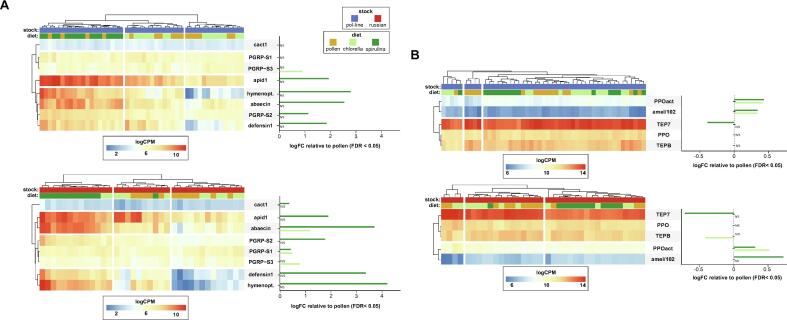
**Algae-substituted diets elicit immune gene expression.** The normalized counts of select (A) humoral and (B) cellular immune genes were first hierarchically clustered by sample and then by gene based on Pearson correlation and then plotted as a heat map. Then, contrasts between algae-fed and pollen-fed bees were calculated and visualized as a bar chart on the right side of each heatmap. Overall, spirulina was more immunogenic and Russian bees had higher immune gene expression. For ease of visualization, transcripts were separately for Pol-line (blue squares along the top) and Russian (red squares along the top) bees.

Given the enrichment of antimicrobial defense genes in spirulina-fed bees, we hypothesized that microalgae would elicit a stronger antimicrobial immune response relative to a natural pollen diet and the response would vary across genetic backgrounds. We chose a subset of previously characterized humoral and cellular immune-related genes for differential expression analyses (Table 2). Overall, the results discussed below suggest Russian bees are immunologically more responsive than Pol-line, especially when fed spirulina (Fig. 6).

Normalized immune gene read counts were plotted as a heatmap with both gene- and sample-wise hierarchical clustering. Notably, Russian bees had more qualitative in-group similarity than Pol-line bees when fed spirulina, as evidenced by better clustering of Russian samples apart from the other two diets (Fig. 4, Fig. 6). This is likely related to the larger immune response to spirulina in Russians and is in-line with the overall atypical transcriptional response in Russians to spirulina. Next, contrasts were calculated in microalgae-fed bees relative to pollen-fed bees to assess microalgae-specific immune stimulation (significant contrasts are plotted as bar plots on the right margin of heatmaps in Fig. 6). Both microalgae species induced humoral and cellular immune gene expression. Regardless of stock, spirulina elicited a stronger humoral immune response than *Chlorella*, though this effect was more pronounced in Russian bees (Fig. 6A). Indeed, *PGRP-S1* and *cactus-1* were significantly upregulated in Russians, but not pol-line. Only one transcript (*PGRP-S3*) was induced by *Chlorella* and not spirulina.

The cellular immune response to algae-containing diets was less stark (Fig. 6B). Interestingly, while *TEP7* is generally highly expressed as indicated by the heatmap, spirulina-fed bees have significantly lower expression compared to pollen-fed. However, microalgae diets resulted in higher expression of two genes involved in melanization (*PPOact* and *amel\102),* which may indicate priming of cellular immunity. Altogether, these data suggest that microalgae may provide the benefit of priming the bee’s immune system in addition to its nutritional benefits.

## Discussion

Bees are important agricultural pollinators and improving their health through nutritional supplementation is a promising avenue to safeguard food security. This study aimed to disentangle genetic and diet effects on bee physiology. This study uncovered nutrigenetic variation in honey bees, an important agricultural pollinator with an associated underlying change in transcriptional program. We leveraged this variation to identify an expanded repertoire of nutritional molecular biomarkers, signatures of altered maturity rate in response to dietary stress, and broad immune activation by microalgae diets that will prove useful in future studies of bee health and nutrition. We outline the implications of these observations below.

### Novel biomarkers reveal nutrigenetic variation in honey bees and trade-off considerations for diet formulation

There is an extensive history of research on the physiological responses of bees to a variety of natural and artificial diets, including pollen, commercial artificial diets and microalgae [20,22,[54], [55], [56], [57]], though comparatively less work has been focused on the genetic basis for variation in response to diet in honey bees [22,54]. Here, we employed a supervised machine learning approach with Recursive Feature Selection to identify an expanded set of biomarkers which accurately predict body weight across genetic backgrounds in starved (i.e., sugar-fed) and PL-fed bees. It is worth noting that while forecasting and prediction with machine learning models sometimes suffers from noisy data with small sample sizes, ensemble methods like Random Forest which uses bagging to aggregate results across randomly generated trees generally perform well on datasets with more features than samples, which is common in omics data [58]. By applying feature selection techniques, like Recursive Feature Elimination (RFE), this “p > n” problem can be further mitigated [39]. Our RFE approach via iterative training of RF regression models and elimination of uninformative features correctly identified two previously described biomarkers, most-notably *Vitellogenin* (*Vg*) (Fig. 2 and Fig. S3). However, we sought to expand the repertoire of nutrition-related biomarkers to assist in rational development of tailored diets as well as marker-assisted selection in breeding programs.

*Vg* is a hormonally-regulated egg yolk lipoprotein, socially coopted by honey bee workers as a nutrient storage protein in nurse bees tasked with caring for developing larvae [59]. Vg titers are positively correlated with immune function [60], resilience to oxidative stress [61,62], and longevity in workers [63] and queens [62]. Additionally, *Vg* mRNA levels are highly predictive of Vg titers in bee hemolymph [64]. Pollen consumption is positively correlated with Vg synthesis in laboratory reared bees [65]. Therefore, *Vg* expression is a particularly effective biomarker for nutritional status in nurse-aged bees. Our Random Forest regression model classified *Vg* as a good predictor of average body weight (Fig. 2C and Fig. S3), suggesting our approach meaningfully identified molecular biomarkers across genetic backgrounds. Interestingly, spirulina-fed bees had lower *Vg* expression than pollen or *Chlorella*-fed bees regardless of stock (Fig. S3), although only Russian bees were lighter when fed spirulina (Fig. 1B). In this experiment, spirulina was nutritious but seemingly suboptimal compared to pollen and *Chlorella*, especially in Russians*.* This contrasts with our previous work in which spirulina lead to higher bodyweight with no effect on *Vg* expression in either stock, although bees consistently consume less spirulina than pollen [22]. The reason for this difference is unclear but underscores the complexity of bee nutrition studies which are inexorably affected by season, genetics and the physiological state of the parental colony [66,67].

Our optimal Random Forest regression model of bee body weight as a function of transcript expression also identified *Jhe* which encodes a negative regulator of JH titers [68]. Task-related genes *like Vg and Jhe* are good indicators of stress in honey bees since colony function relies on age-related task specialization from nursing to foraging. Indeed, both *Vg* and *Jhe* have been shown to be responsive to immune and heat stress [69] and nutritional stress [70]. *Jhe* expression is down regulated in forager bees which is associated with reduced longevity [69]. Taken together with lower *Vg* expression, one would predict that bees fed spirulina matured precociously, which is negatively associated with both individual and colony longevity [71,72]. This is salient in light of lower expression of *LOC411893* (aka*, sad*) which encodes for a mitochondrial P450 enzyme involved in the final steps of ecdysteroid synthesis [73], as ecdysteroid synthesis and JH synthesis have complex cross-talk with one another during coordination of task-related development [74]. There is evidence that while elevated JH tiers suppress vitellogenesis, ecdysteroids may prepare worker tissues to uptake Vg, implying contrasting functions during task development [75]. In other words, lower ecdysteroid biosynthesis would be expected in the case of elevated JH titers due to reduced *Jhe* expression. Downregulated *hex70a* expression in Spirulina-fed bees may be counterfactual to this line of thought, as *hex70a* transcripts are generally high early in life and degrade in aging honey bee workers [76]. However, *hex70a* transcripts are not known to respond to immune challenge, the protein hex70a degrades quickly following immune challenge [77] and thus reduced expression likely indicates physiological stress. These four biomarkers alone suggest a decoupling between canonical biomarkers (*Vg* and *Jhe*) and body weight in Pol-line bees, while they are likely more predictive in a comparatively sensitive Russian bee. Their expression patterns also strongly underscore the trade-offs associated with immune stimulation by ingestion of Spirulina. But taking the full panel into consideration reveals that *Chlorella* may be an especially good dietary choice for Russian bees.

Two of our biomarkers (*Glob1*, and *slif*) have no clear functional associations to the JH-Vg endocrine axis, but encode for metabolically important proteins localized to the fatbody. *Glob1* is a good functional biomarker candidate as it encodes for an intracellular fat body-expressed globulin important for O_2_ homeostasis in insects [43,78]. This implies that *Glob1* serves a respiratory function in the insect fat body [79] which are highly metabolically active tissues and sites of energy mobilization. Higher *Glob1* expression in bees fed *Chlorella* may therefore indicate higher levels of respiration and energy mobilization, indicating that *Chlorella* may be an exceptionally good nutritional source. On the other hand, *slif* encodes an amino acid transporter involved in the regulation of TSC/TOR signaling in the insect fat body. The expression of *slif* is strongly downregulated in nutritionally restricted *Drosophila* which stunts growth via repression of PI3K signalling, thereby acting as a fatbody-localized nutrient sensor [80]. Therefore, it stands to reason that a general nutrient sensor like *slif* would show little variation between PL-diet fed bees, but perhaps lowers its utility for comparison of PL-diets. The combination of weight measurements and transcriptomic approaches enabled us to capture that *Chlorella* may be an exceptionally good diet and that genetic variation plays a key role in nutritional response. Altogether, we identified known and novel biomarkers which require further validation in field studies to confirm their generalizability to the colony setting.

### Microalgae are a functional food with immune-stimulatory properties

In this study all protein/lipid containing diets resulted in higher immune gene expression. This is unsurprising as the immune system exacts a high demand of both macro- and micronutrients [81]. In fact, the amount and complexity of a pollen-based diet both positively correlate with improved immunocompetence in honey bees [15,82]. We hypothesized that both microalgae would elicit an immune response beyond pollen due to the cell wall-associated polysaccharides associated of both spirulina and *Chlorella* [83] which have proven to be immunomodulatory in a variety of vertebrate and invertebrate organisms [[84], [85], [86], [87]].

The role of cell wall-associated is substantiated by the specific upregulation of *PGRP-S3/SA* by *Chlorella* consumption as PGRP-S3 specifically binds to N-acetylglucosamine moieties present in the cell wall of *Chlorella vulgaris* [88,89]. Indeed, both stocks had higher expression of several immune genes when fed microalgae diets. Overall, spirulina was clearly more immunogenic than Chlorella and Russian bees were more responsive to its effects. This higher immune activation, including *ago2* expression could partially explain why Russian bees had significantly lower DWV loads. Higher immune responsiveness might also explain lower DWV-B in Russian colonies despite similar mite levels to Pol-line colonies [31]. In conjunction with the signature of stress response gene transcription, one would conclude that the lower body weight in Russian bees is at least partially due to some toxicity of spirulina biomass. Intriguingly, Pol-line bees showed some indication of a stress response but with a smaller magnitude, likely due to genetic variation in sensitivity to immune activating signals such as peptidoglycans or rate of immune senescence [90,91]. In any case, the immunogenicity of spirulina is underscored by the fact that immune challenge has previously shown to reduce the expression of both *Vg* and *Jhe* in nurse-aged bees [90]. The lower expression of biomarkers in bees fed Spirulina (Fig. 2, Figure Ss) implies there is a cost associated with immune stimulation, as observed previously in bees [77,[92], [93], [94], [95], [96]]. However, we recently reported that a basal commercial diet substituted 20 % w:w with Spirulina powder induces an immune response which is associated with enhanced bacterial clearing and longevity [32]. Therefore, there is likely an optimal proportion of spirulina diet substitution to achieve a protective immunological effect through hormesis [97] whereas here all proteinaceous biomass was spirulina powder.

The nutrient content and immunostimulatory properties of spirulina as well as its sustainable production make it an attractive candidate for functional feed additives. Regarding its scalability, we previously found that feed substituted with spirulina benefited honey bee colony-level performance and health metrics in a commercial beekeeping operation [98]. However, additional benefits of algae feeds could be obtained through genetic modification [99]. Engineered feed could enable fine-tuning the biomass nutrient profile [100] or leveraging microalgae cells as biomanufacturers of functional biomolecules [101] such as edible vaccines [102]. For example, we previously demonstrated that *Synechococcu*s, a related cyanobacterium (blue-green alga), could be engineered to produce virus-specific double stranded RNAs (dsRNAs) and effectively function as edible antiviral treatments for bees[24]. This is especially timely as ingested dsRNAs were recently demonstrated to be effective at reducing survivorship of the ectoparasitic Varroa mite in the lab [103,104] and in the colony [105], via transfer of dsRNA from the hemolymph of the bee to the mite [106]. The modifiability and immune stimulating properties of spirulina in conjunction with the aforementioned superior nutritional qualities of *Chlorella* could be close to an ideal nutritious package for delivering therapeutics to bee colonies. By leveraging the rapidly growing field of genome editing and synthetic biology, tailor-made nutritiously ideal packages that co-deliver targeted therapeutics for colony management are becoming increasingly plausible [25,26].

### Russian bees have a contracted transcriptional response and exhibit transcriptional signatures of delayed behavioral maturation

The shared transcriptional response to a PL diet (Fig. 4A) that we observed is broadly similar to what has been previously observed in other organisms relative to starved animals in terms of the number of DEGs and enriched GO terms [107]. It is interesting, however, that Russian bees had a strikingly contracted transcriptional response with lower intra-individual variability relative to Pol-line bees (Fig. 4). It is tempting to speculate that the enrichment for differential expression of chromatin structure-associated proteins and lncRNAs are in some way associated with this contracted response, and that lncRNAs may be a major driver in the nutrigenetic variation between the two stocks. Indeed, of the top 20 genes whose expression accurately classified bees as Russian or Pol-line, 7 of them are predicted lncRNAs. There is precedent for this hypothesis as honey bee lncRNAs have been implicated in tissue identity and the response to viral infection [108] as well as playing a potential role in coordinating gene regulatory networks during infection with *Nosema ceranae*, a microsporidian pathogen [109]. So, it seems plausible − if not likely − that lncRNAs also contribute to gene regulation in response to nutritional perturbations.

Russian bees appeared to have a transcriptional signal of delayed behavioral maturation relative to Pol-line bees in this study. This may contribute to the reduced number of genes which are differentially expressed at our chosen timepoint. However, lower expression of *JAHMT,* which was identified by our SVM model as discriminating between stocks, strongly implies a relative delay in behavioral maturation and/or senescence in Russian bees. Lower JAHMT should result in commensurately lower juvenile hormone (JH) at the time of sampling [110,111]. An increase in JH titers accompanies a behavioral switch from nursing to foraging in honey bees [52]. The heavier weight of Russian bees could also be explained by this delay in maturation, as the switch to the forager phenotype coincides with energy mobilization from the fat body which is marked by elevated *AKHR* expression, which was downregulated in Russian bees [112]. Mutant flies lacking *AKHR* activity show increased resilience of starvation and higher body mass [113], while elevated *AKHR* expression is associated with starvation susceptibility and carbohydrate mobilization in worker bees [114]. It is worth noting that if Russian bees had matured more slowly in this experiment, one would predict a larger immune response to spirulina relative to Pol-line bees, as the immune systems of older bees senesce leading to smaller immune gene responses [115]. We propose that these transcriptional signatures underly fundamental differences in the strategy of social integration of nutrition in Russian and Pol-line bees. For example, we would predict the more rapid energy mobilization of the Pol-line bee to be associated with a faster colony build-up, which is one of the attractive features of this stock. However, slower pace of energy mobilization in the Russian bee led to heavier bees which showed a transcriptional signal of starvation resilience, which may be an attractive feature for some growers. Understanding the molecular mechanisms underpinning colony-level behaviors could further aid in developing tailored feeding regimes which meet the dynamic nutritional needs of managed bee colonies [116].

## Conclusion

Here, we present evidence that two microalgae species have complimentary benefits as functional food additives for honey bees. These findings open the possibilities to augment bee disease resistance through nutritional interventions. While *Chlorella* was particularly nutritious, the immunogenicity spirulina may confer unique benefits to pathogen resistance. We also leveraged observed nutrigenetic variation to uncover alternative nutrient assimilation strategies in two Varroa mite resistant stocks, which will help inform management decisions and feed development for healthier colonies. Lastly, the comparative sensitivity of Russian bees to trade-offs associated with immune stimulation is an important consideration in the design and deployment of engineered biotherapeutics. Effective management strategies need to toe the line between generally effective and targeted without conferring cost to the colony. Nutritional needs and physiological effects of the protein/lipid source of our premiere agricultural pollinator inevitably vary across time, space, and genetic background.

## Funding

This work was supported by Agriculture and Food Research Initiative grant no. 2021–67013-33556 from the USDA National Institute of Food and Agriculture. This research was supported in part by an appointment to the Agricultural Research Service (ARS) Research Participation Program administered by the Oak Ridge Institute for Science and Education (ORISE) through an interagency agreement between the U.S. Department of Energy (DOE) and the U.S. Department of Agriculture (USDA). ORISE is managed by ORAU under DOE contract number DE-SC0014664.

## Declaration of competing interest

The authors declare that they have no known competing financial interests or personal relationships that could have appeared to influence the work reported in this paper.
