# Supplementary material for: Honey bee nutritranscriptomics reveals key insights towards precision nutrition
Source: J Adv Res. 2025 Sep 30;84:95–108. doi: 10.1016/j.jare.2025.09.050 (PMC13227215; doi:10.1016/j.jare.2025.09.050)
Supplement: Supplementary Data 1 [file mmc1.pdf]

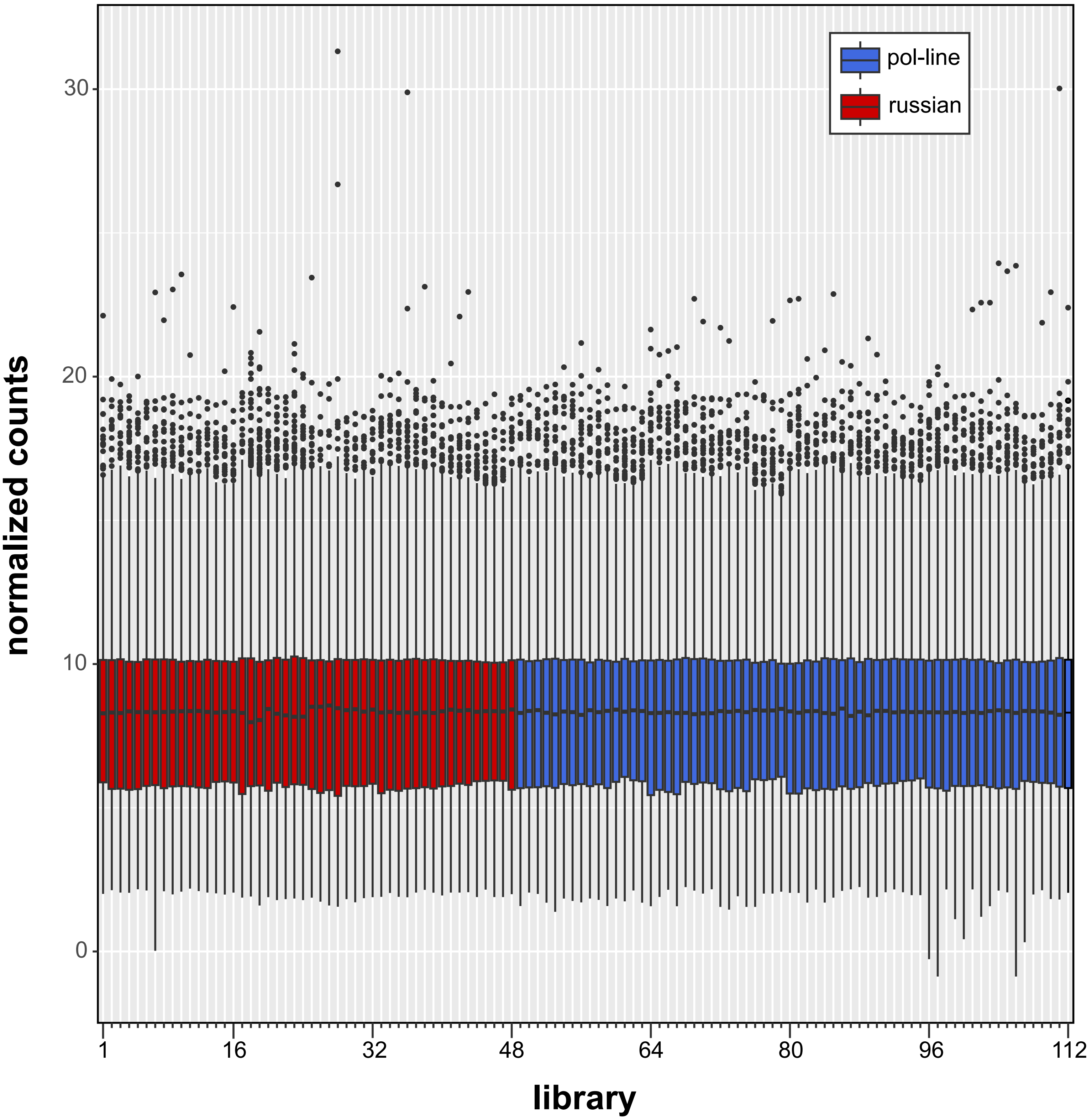

Figure S1. Normalized transcript counts after filtering for low-read transcripts plotted as boxplots. All libraries display uniformity, demonsrrating adequate normalization

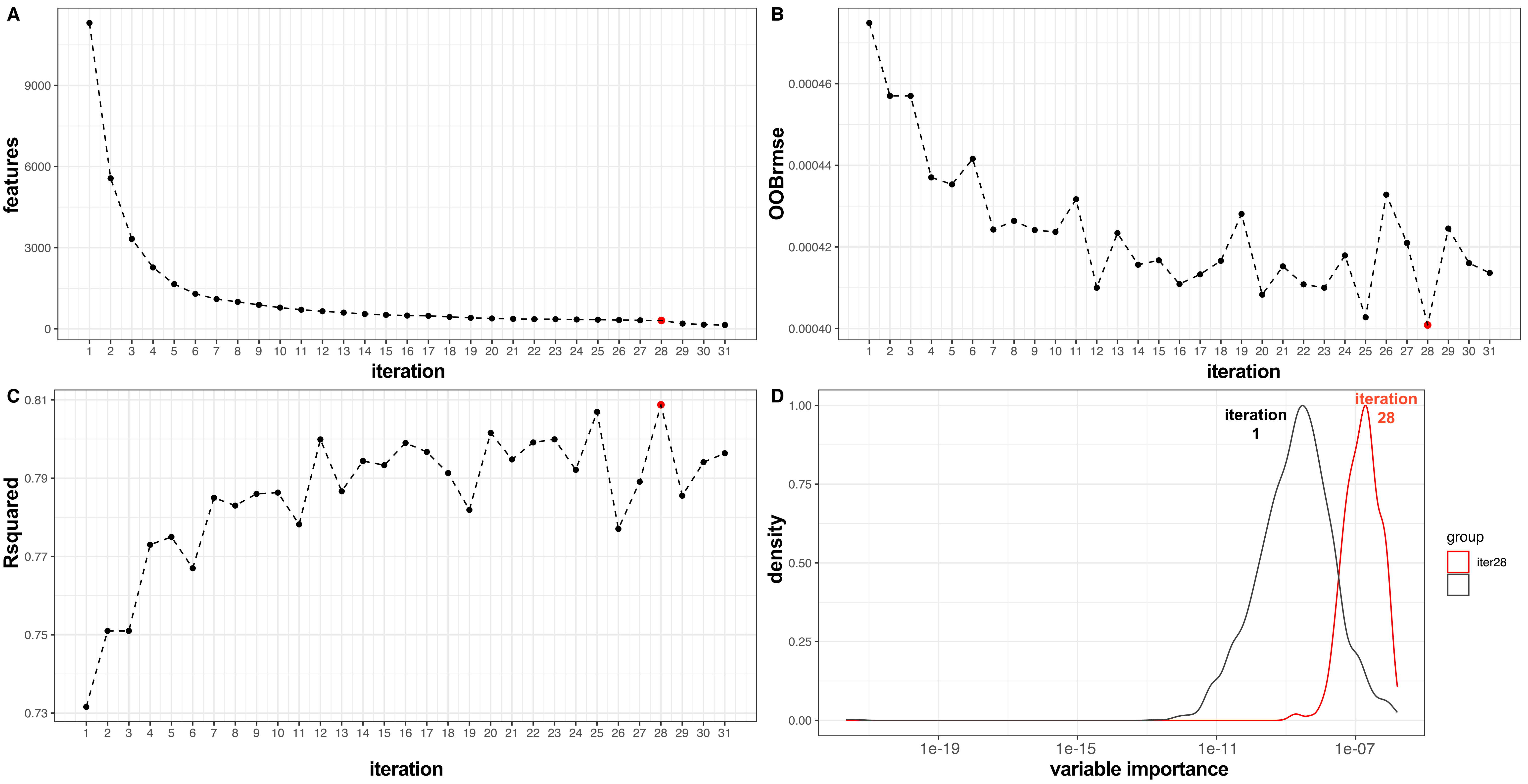

Supplemental Figure 2. A random forest regression model was used to predict bee body weight as a function of normalized transcript abundance (Tables S3 and S4). A Recursive Feature elimination approach was used to identify the optimal number of features for model performance as measured by OOB MSE minimization. After each iteration of model building and tuning, trancripts showing an importance value of 0 or less wre removed, generating a smaller set of features for the next iteration. As the number of features per iteration (Panel A) were reduced, models generally performed better as measured by a reduction in (Panel B) OOB RMSE and and increase in regression model (Panel C) R-squared. OOB RMSE was the smallest in the 28th iteration, indicating 303 transcripts were the optimal number of features to build the model. As a corollary, the average variable importance values of features were higher in iteration 28, indicating greature contribution to model prediction.

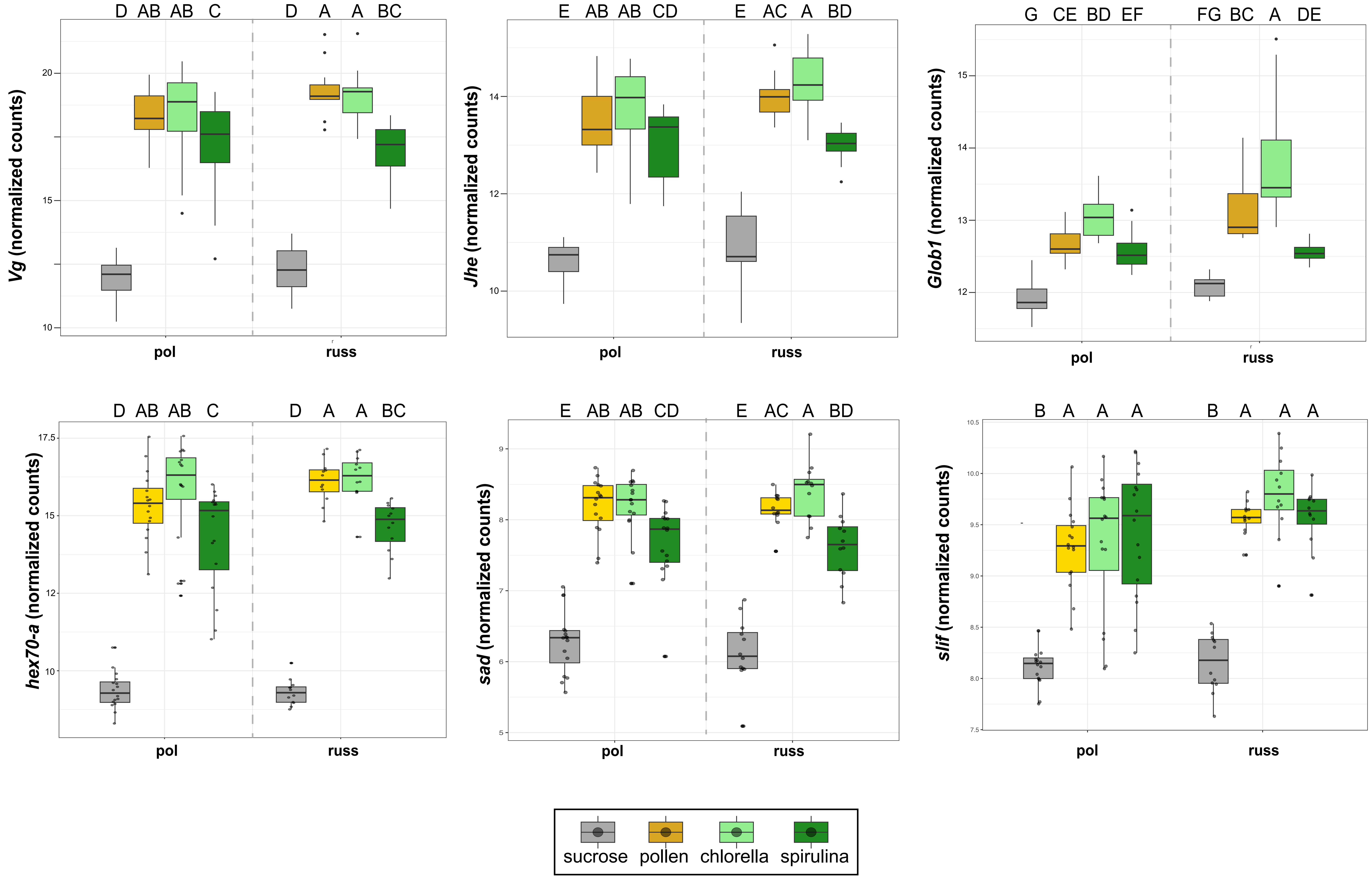

Supplemental Figure 3. A random forest regression model was used to predict bee body weight as a function of normalized transcript abundance (Tables S3 and S4). A Recursive Feature elimination approach was used to identify the optimal number of features for model performance as measured by OOB MSE minimization. After each iteration of model building and tuning, trancripts showing an importance value of 0 or less wre removed, generating a smaller set of features for the next iteration. The optimal model included 308 transcripts for body weight prediction. We further pared down those features by assessing correlation with body weight, and then chose 6 of the top transcripts for further investigation in the context of this study as biomarkers for bee nutrition studies.

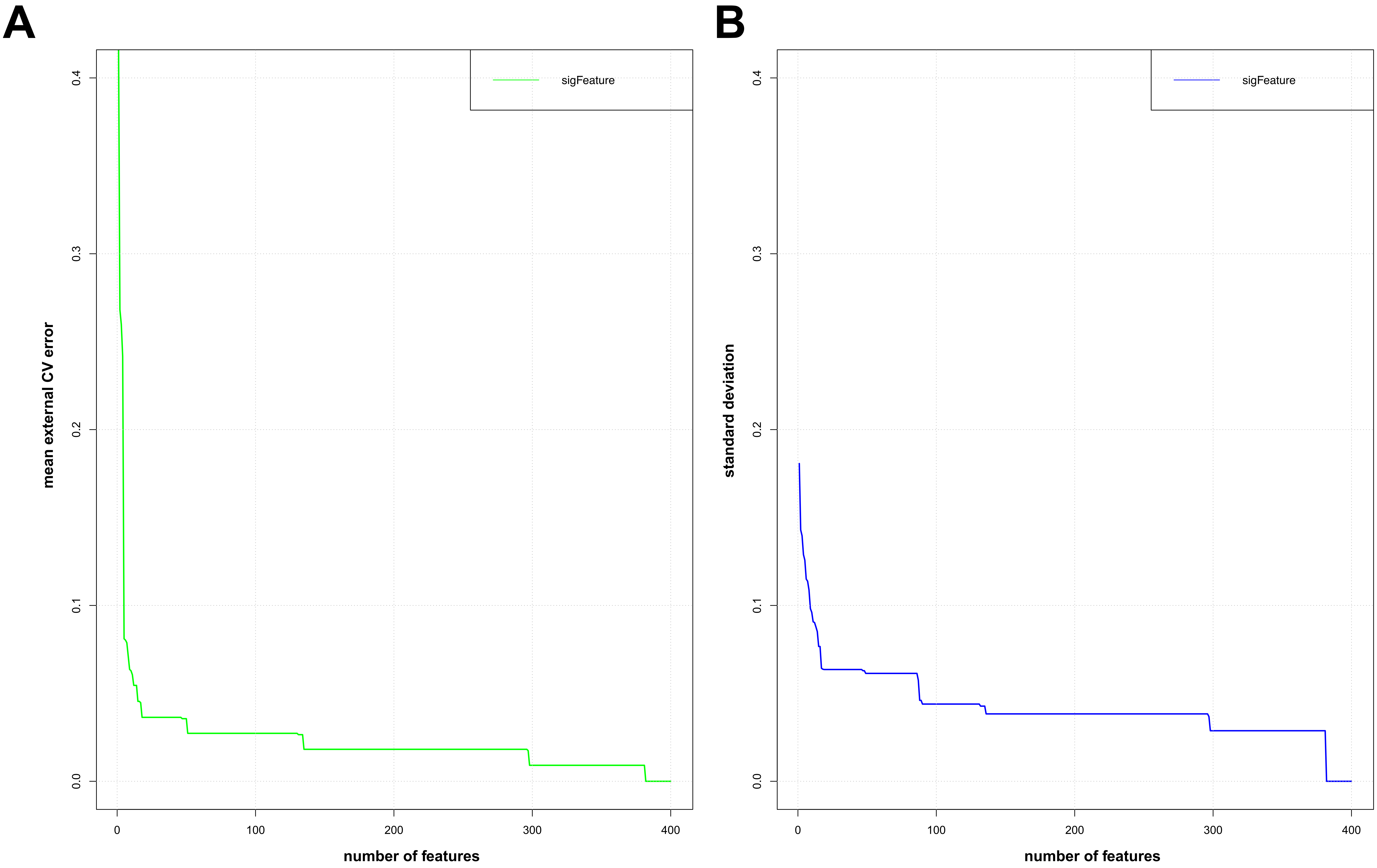

Figure S4. 10-fold cross validation was applied to assess SigFeature SVM model performance in identifying transcripts with the most discriminatory power between the two classes of samples: Russian and Pol-line genetic stock. The model identified nearly 400 transcripts as the optimal number of features for classifying samples as mean CV error (Panel A) and the standard deviation of those errors (Panel B) reached zero.

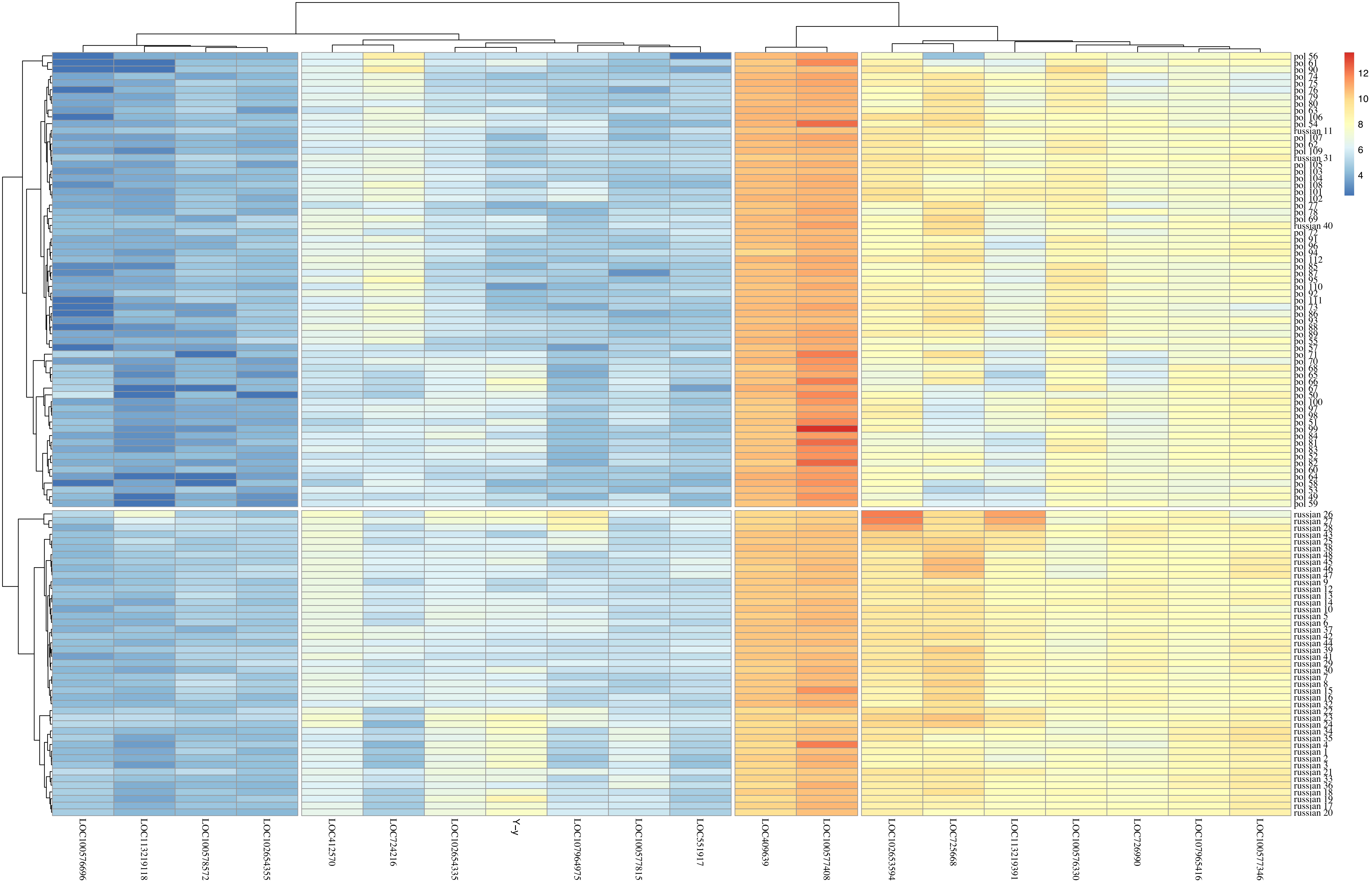

Figure S5. 10-fold cross validation of the SVM model built to predict bees as either Russian or Pol-line identified 400 transcripts with maximum discriminatory power between samples. For ease of visualization and intepretation, the top 20 genes (which stil lshowed high discriminatory power) were chosen for further consideration. Normalized transcript counts were plotted with hierarchical clustering by Pearson correlation by row and by column (i.e., by sample similarity and then by transcript expression similarity). This revealed two distinct sample blocks roughly split by genetic stock, excepting russian samples 11, 31 and 40. Furthermore, this identified 4 groups of transcripts with the highest similarity of expression between them. See Table S11 for accession, description and direction of differential expression for each transcript.

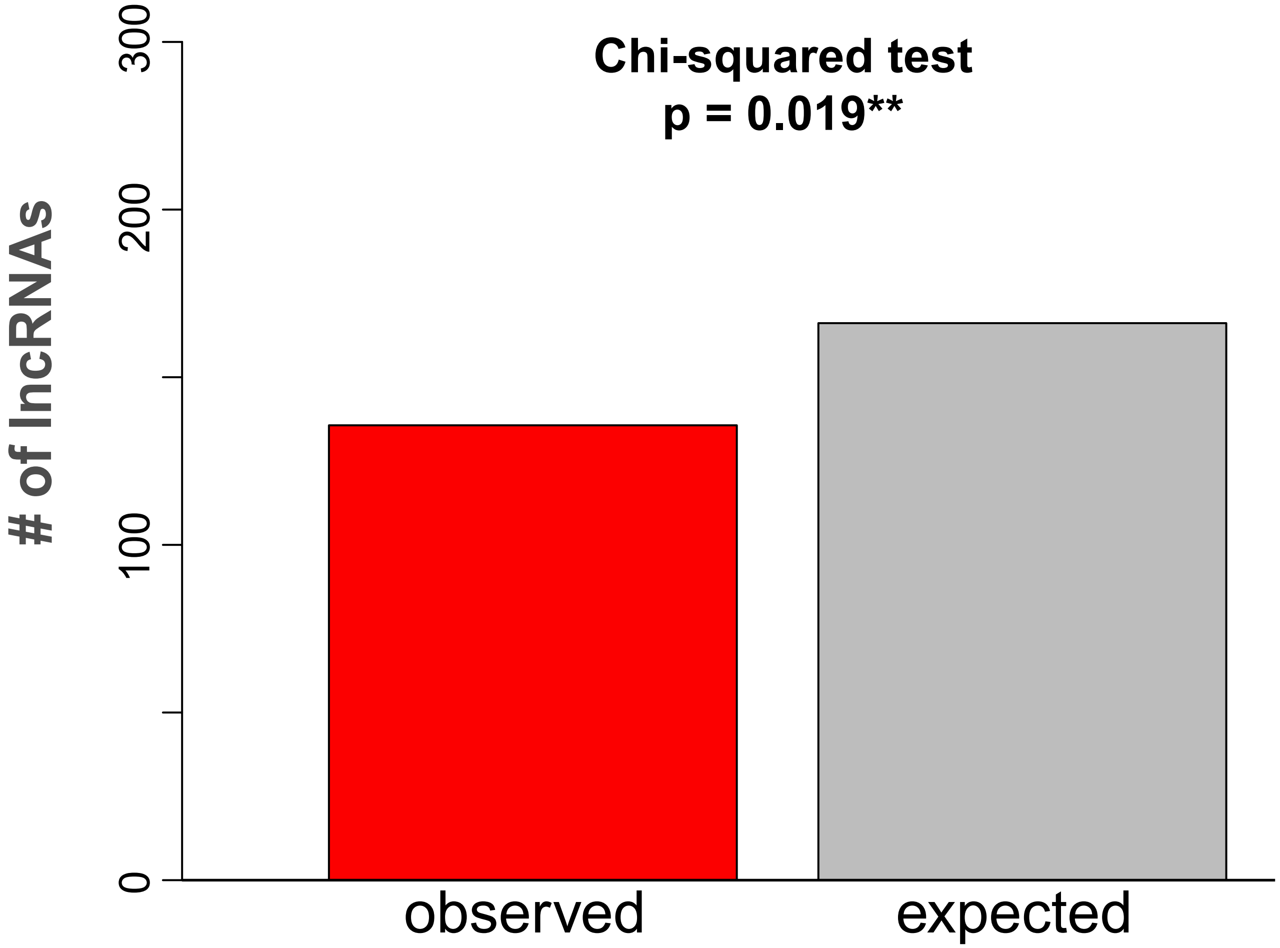

Figure S6. Observed versus expected number of lncRNAs in pol-line unique DEGS correspecding to column 2 on Table S13 and the blue Pol-line portion on Figure 4A.

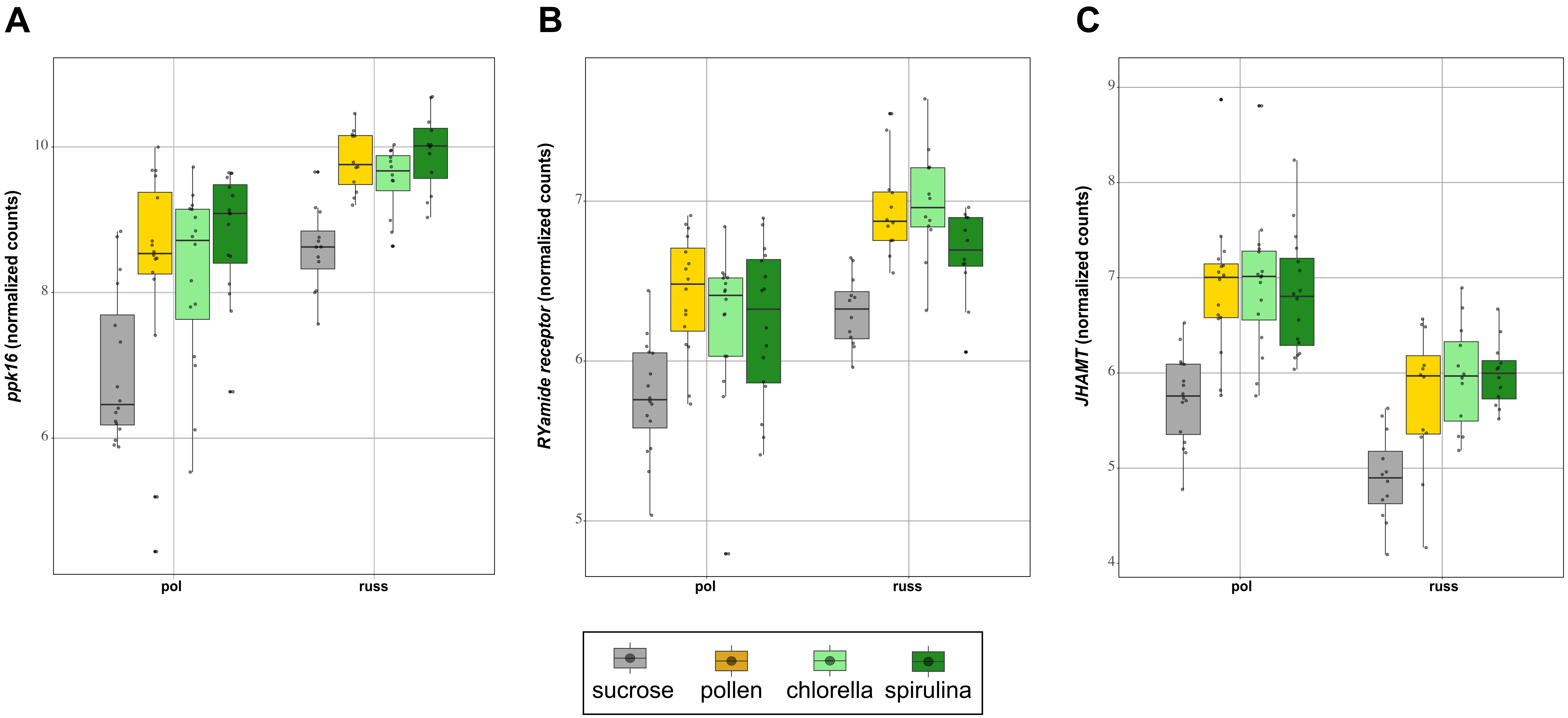

Supplemental Figure 7. Of the 20 transcripts identified as having high discriminatory power in classifying bees as either Russian or Pol-line, 3 of them were chosed for further consideration due to known or predicted functions. Overall, ppk16 (panel A) and RYamide receptor (panel B) showed higher expression in Russian bees regardless of diet, while JHAMT (panel C) showed significantly lower expression than Pol-line bees regardless of diet. See the Results and Discussion sections, Table 11 and Table S15 for descriptions of function and statistics for expression.

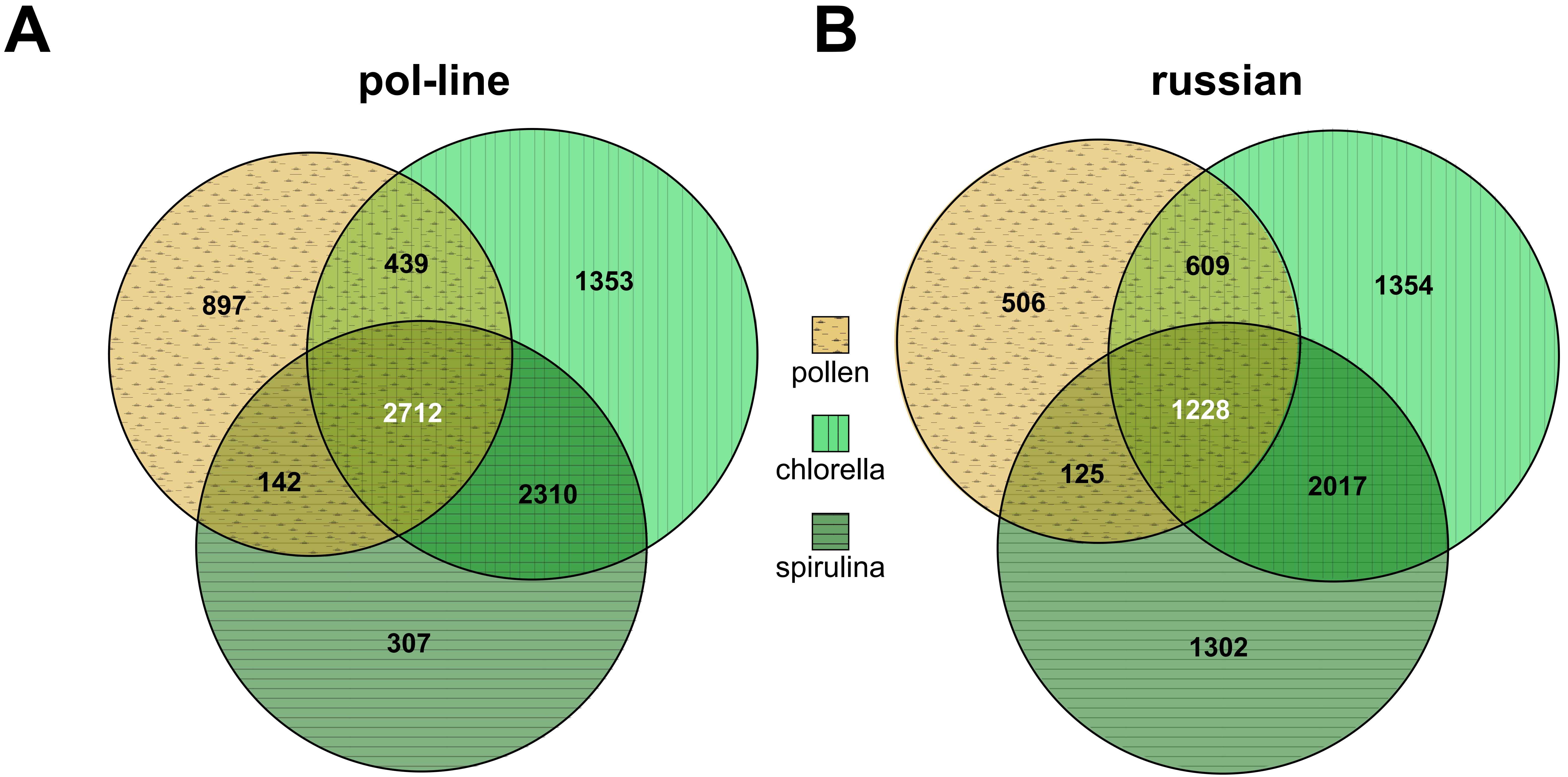

Supplementary Figure 8. Venn diagram showing DEG overlap between diets within (A) Pol-line and (B) Russian bees. The background was the entire list of 12140 genes in the honey bee transcriptome.

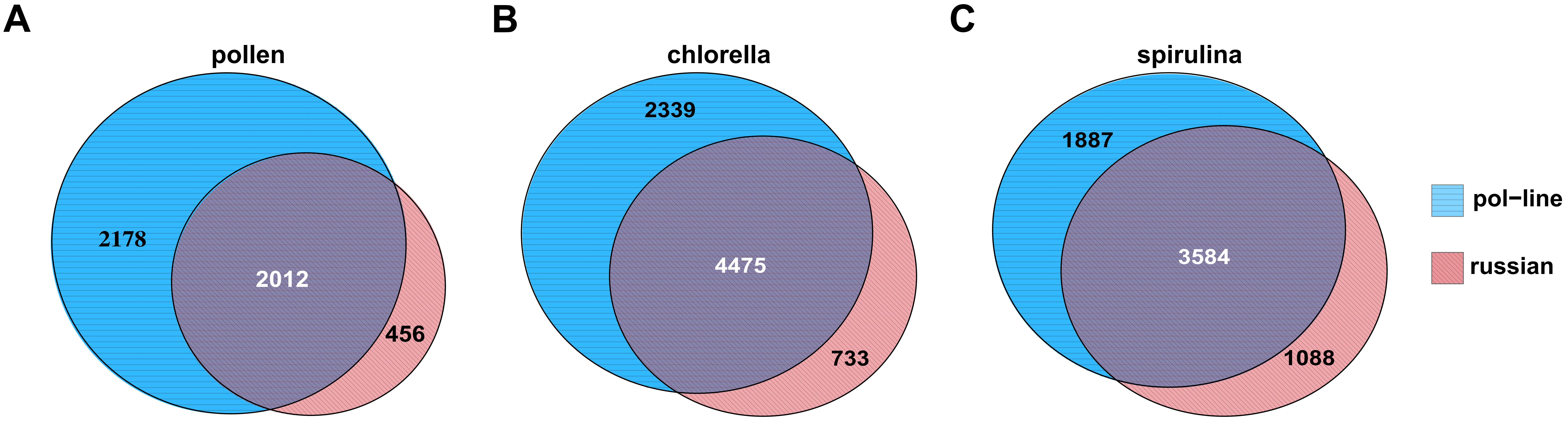

Supplementary Figure 9. Venn diagrams of DEGs within diets. The background was the entire list of 12140 genes in the honey bee transcriptome.
